# CCT6A functions as promising diagnostic biomarker and promotes cell proliferation in colorectal cancer

**DOI:** 10.7150/jca.98901

**Published:** 2024-09-16

**Authors:** Jianxing Ma, Qiuya Wei, Lili Zhang, Fengyao Sun, Wen Li, Ruihang Du, Mingchan Liu, Siyuan Yan, Chen Wang

**Affiliations:** 1 Department of General Surgery, the Second Hospital of Lanzhou University, Lanzhou 730000, China.; 2 Precision Medicine Laboratory for Chronic Non-communicable Diseases of Shandong Province, Institute of Precision Medicine, Jining Medical University, Jining 272067, China.; 3 Department of Oncology, the People's Hospital of Jiaxiang, Jining 272499, China.

**Keywords:** CCT6A, colorectal cancer, cell proliferation, cell migration, bioinformatics analysis

## Abstract

**Background**: Chaperonin-containing tailless complex polypeptide 1 subunit 6A (CCT6A) is mainly located in the cytoplasm and considered to be involved in various biological processes in tumors. However, its function and the intrinsic mechanism need to be further elucidated.

**Methods**: Multi-omics analysis was used to evaluate the correlation between CCT6A expression and prognosis of patients, as well as its immune value. CCT6A was knockout by CRISPR-Cas9, and overexpressed by transfecting plasmids in colorectal cancer (CRC) cells. Cell proliferation was analyzed by MTS, EDU staining and colony growth assay, and cell migration was monitored by wound healing assay and Transwell assay. The phosphor-kinase array kit and immunoblotting assay was utilized to explore the potential molecular mechanisms.

**Results**: CCT6A was highly expressed in multiple tumor tissues and significantly correlated with the prognosis of patients. It was also associated with the immune infiltration, immune correlation and prognosis in CRC. CCT6A was highly expressed in CRC biopsies as well as fresh CRC tissues. Meanwhile, knockout of CCT6A reduced cell proliferation, cell cycle and cell migration. On the contrary, overexpression of CCT6A exhibited the opposite phenotypes. Moreover, we identified that HSPD1 and non-phosphorylated P53 were highly increased in CCT6A overexpressed cells, which are involved in regulating tumorigenesis.

**Conclusions**: Therefore, CCT6A positively regulated cell proliferation/migration in CRC cells, and suggesting CCT6A has a high immunological value and is associated with CRC progression, which makes it a potential therapeutic target for CRC.

## Introduction

Globally, colorectal cancer (CRC) is the third most common cancer and the second leading cause of death, accounting for approximately 10% of malignancies and cancer-related deaths diagnosed each year 1, 2, making it a significant burden on society 3. The majority of patients have progressed to the intermediate- and advanced-stage when they exhibit severe clinical symptoms, which is one of the reasons for the poor therapeutic effect of CRC 4. Therefore, it is critical to explore novel therapeutic targets and strategies, as well as investigate novel early diagnostic biomarkers for CRC.

Chaperonin-containing tailless complex polypeptide 1 subunit 6A (CCT6A) belongs to the Group II chaperone protein complex, which consists of two identical stacked rings, each containing eight distinct proteins. Several studies indicated that it is involved in modulating cell proliferation, migration, and invasion in cancer cells 5-7. By analyzing public database data and clinical data of 220 patients with surgical prostate cancer, Song *et al.* found that high expression of CCT6A increased Gleason score and pathological T stage and reduced disease-free survival (DFS) 8. Meanwhile, knockdown of CCT6A reduced cell proliferation, cell viability and promoted apoptotic cell death in osteosarcoma cells 9. Furthermore, a long-term follow-up study showed that high expression of CCT6A was associated with poor DFS and late TNM stage in patients undergoing gastric cancer surgery 10. Ma *et al.* reported that high expression of CCT6A and MYH9 was associated with shorter overall survival (OS) and DFS in patients with CRC liver metastasis, which suggests the potential prognostic value of CCT6A in this group of patients 11. As a member of homologous box proteins which are essentially transcriptional regulatory factors 12. HOXB2 increases the proliferation and invasion ability of colon cancer cells by up-regulating the expression of CCT6A 7. On the contrary, it has been reported that the expression of CCT6A is negatively correlated with patients' DFS and OS in cervical cancer 13. CCT6A is also believed to be related to immunity. By dynamic network biomarker (DNB), CCT6A was identified as a biomarker for the pre-exhausted T cells subpopulation in CRC. As a core DNB gene, TUBA1B expression is triggered by CCT6A and is involved in CD8^+^ T cell exhaustion 14. However, more comprehensive bioinformatics analysis and extensive experimental validation are necessary to fully understand the role of CCT6A in affecting cell proliferation/migration and its immune value in CRC.

Recent years, the tumor immune microenvironment (TIME) of CRC has been an important contributor to disease biology and has been playing an increasingly important role in the cancer therapy and intervention 15. Owing to the diversity of immune cell populations in different subpopulations of CRC, it has been discovered that the immune signature of TIME has predictive and prognostic value for patients. Tremendous changes have emerged in the clinical treatment concept and methods of CRC, which were attributed to the advancement of research on molecular mechanisms, cellular characteristics as well as the increasingly extensive application of immunotherapy in solid tumors 16. Monoclonal antibodies that target key immune checkpoints, such as antibodies against programmed death 1 (PD-1) and cytotoxic T lymphocyte antigen 4 (CTLA-4), have been shown to be clinically effective in treating patients with high microsatellite instability and mismatch repair deficiency (dMMR-MSI-H) 17. However, the utilization of immune checkpoint inhibitors (ICIs) in other subtypes of CRC is limited and needs further investigation 18.

In the current study, we showed that CCT6A is highly expressed in several cancers and associated with OS through bioinformatics analysis. Beyond being related to prognosis, CCT6A also possessed immune value in CRC. Utilizing a human CRC tissue microarray and patients' surgically resection tissues, we verified that CCT6A is highly expressed in the cancer tissues. In the CRC cells, CCT6A overexpression promoted cell proliferation and migration by several detection methods, while knockout of CCT6A presented opposite phenotypes. Taken together, we found that CCT6A is highly expressed in CRC, has a high immune value, and positively modulates cell proliferation and migration.

## Materials and Methods

### Chemicals and antibodies

The Attractene transfection reagent (301005) was purchased from QIAGEN. The Cell Cycle Detection Kit (KGA512) was purchased from KeyGEN BioTECH. IgG-two-step immunohistochemistry kit (SV0004), hematoxylin staining solution (AR1180-1) and DAB staining solution (AR1027-3) were obtained from BOSTER. The primary antibodies of CCT6A (19793-1-AP), Flag (20543-1-AP), and Tubulin (66240-1-Ig) were purchased from proteintech.

### Cell culture and immunoblotting analysis

Human CRC cell lines HT29 and SW480 were cultured with DMEM (GIBCO) supplemented with 10% fetal bovine serum (GIBCO) and antibiotic (BIOODIN). The total proteins were extracted by RIPA buffer containing protease inhibitors. Then the proteins were isolated by electrophoresis in prefabricated SDS-PAGE gel (12%) and transferred to PVDF membrane. Immunoblotting was performed with the corresponding primary antibody and secondary antibody coupled with horseradish peroxidase, followed by detection with an enhanced chemiluminescent solution (Beyotime). For plasmids transfection, cells of 60% confluency were transfected with indicated plasmids using Attractive Transfection Reagent according to the manufacturer's handbook. The overexpressed plasmid of CCT6A (G123304) was purchased from Fenghui Biotechnology, along with the control empty plasmid. The CRISPR-CAS9 knockout plasmids for CCT6A (YKO-RP003-hCCT6A: 5'CGGTCAACATCAGCGCAGCGCGG3', YKO-RP003-hCCT6A: 5'GGTCAACTTCAGCGCAGCGCGGG3') were got from Genai Biotechnology.

The CRC tissues were obtained from patients who underwent surgical treatment at the Second Hospital of Lanzhou University in 2021-2022 (without obvious complications, malignant, T equal or greater than 3), and then the total proteins were extracted and performed immunoblotting assay with indicated antibodies. The patients' information is listed in **Supplementary Table 1**.

### RNA extraction and qPCR analysis

The total cellular RNA was extracted by using TRIzol (Invitrogen) reagent according to the manufacturer's instructions, and 1ug of RNA was reverse transcribed by incubating at 37 °C for 15 min using EasyQuickMasterMix (CWBIO). The primer sequences for amplification were as follows:

qPCR was initiated with a denaturation at 95 °C for 10 min. The cycle program was 95 °C (15 s), 60 °C (45 s) and 72 °C (1 min) for up to 40 cycles. Finally, the data were calculated based on the internal control of β-actin.

### Cell viability assay (MTS)

The cells were divided into 96-well plates (5000-10000 cells per well), and after the overnight culture, the medium was changed to the phenol red-free medium. Following added 10 µL MTS/PMS (20:1) per well, the cell activity was detected at 492 nm absorbance by microplate reader.

### Colony growth assay

The cells were split into 12-well plates at a concentration of 100 cells/mL and cultured with complete medium for 14 days. Cells were fixed with 4% paraformaldehyde (PFA) for 15 min, and then stained with Giemsa dye, the pictures were taken and the Image J software was used to count the number of clones.

### EDU staining assay

The cells were divided into 24-well plates and cultured overnight. Then EDU (1:1000) was added and incubated for 1 h. Follow-up experiments were carried out according to the manufacturer's instructions (C0078S, Beyotime Biotechnology). Pictures were taken by fluorescence microscopy and analyzed by Image J.

### Wound healing assay

5 × 10^4^ cells were added into each well of IBIDI Culture-Insert (80469) and culture overnight. The Culture-Inserts were removed vertically, and the cells were washed with PBS. Low serum medium (1% FBS, 1% antibiotics) was added to each well. Then the wound closure was captured by microscopy. The migration was determined using the Photoshop software as an average closed area of the wound relative to the initial wound area.

### Transwell assay

Matrigel diluted in pre-cooled serum-free medium (1:8) was added to the upper chamber of Transwell and incubated for 1 h. 5 × 10^4^ cells in serum-free medium (400 μL) were added into the upper chamber, and 800 µL complete medium (10% FBS, 1% antibiotic) was added to the lower chamber. After culture for indicated period, the cells on the top side of the membrane were removed by a swab. Then the left cells were fixed by 4% PFA, stained with crystal violet (at room temperature, 2-3 h), and washed with PBS for 3 times. Finally, images were taken under the microscope and counted by Image J software.

### Immunochemistry (IHC)

The human tissue microarray (D100Co01) was purchased from Ernan Biotechnological Company (Xi'an, China). Following baking, dewaxing, hydration, and antigen retrieval, the Rabbit/mouse IgG-two-step immunohistochemistry kit (SV0004, BOSTER) was employed according to the manufacturer's instructions, utilizing a primary antibody diluted at a ratio of 1:100. Subsequent to DAB (AR1027-3, BOSTER) color development, hematoxylin staining, dehydration, and mounting, the slides were scanned using 3D HISTECH technology. The IHC results were quantitatively evaluated by calculating the product of two factors: the percentage of positively stained cells (scored as 1, 2, 3, and 4, corresponding to 0-25%, 26-50%, 51-75%, and 76-100% positivity, respectively) and the staining intensity (scored as 1, 2, and 3, representing low, moderate, and high intensity, respectively).

### Transcriptional, expressional and survival analysis of CCT6A

Transcriptional levels of CCT6A were analyzed in 33 tumors through using TIMER2 database and GEPIA database (http://gepia2.cancer-pku.cn), and CCT6A expression levels were analyzed by CPTAC proteome dataset in UALCAN database. Then, the IHC staining of four kinds of tumor and normal tissues were obtained from HPA (https://www.proteinatlas.org/) database. Additionally, the survivals of them were analyzed by using the GEPIA database and the transcriptional levels of CCT6A in COAD dataset of TCGA database was analyzed again by R 4.2.2.

### GO analysis, KEGG and GSEA analysis

Relevant COAD and READ datasets were downloaded from TCGA official website (https://www.cancer.gov/ccg/research/genome-sequencing/tcga) and performed GO, KEGG, GSEA analysis with R 4.2.2.

### TIME differential analysis, immune cell infiltration analysis

The R packages "limma" and "estimate" were used for TIME differential analysis of CCT6A in COAD and READ datasets. Moreover, the CIBERSORT algorithm was used in R 4.2.2 for immune cell infiltration and correlation analysis.

### Analysis of immune checkpoint correlation and immunotherapy

The immune checkpoint correlation analysis was performed by using R 4.2.2, and the correlation analysis pFilter was set to 0.001. Additionally, Immunotherapy analysis was performed by using the "limma" and "ggpubr" packages in R4.2.2 after immune score data was downloaded from the TCIA database (https://tcia.at/).

### Statistical analysis

The data with normal distribution are shown as mean ± SD. The significance of statistical differences between the two groups was assessed by using the Student t test. Multigroup comparisons of the means were performed by one-way analysis of variance with a post hoc Student-Newman-Keuls test. P < 0.05 was regarded as statistically significant. Most of data were acquired from three independent experiments.

## Results

### Expression of CCT6A in pan-cancer

**Figure 1:**
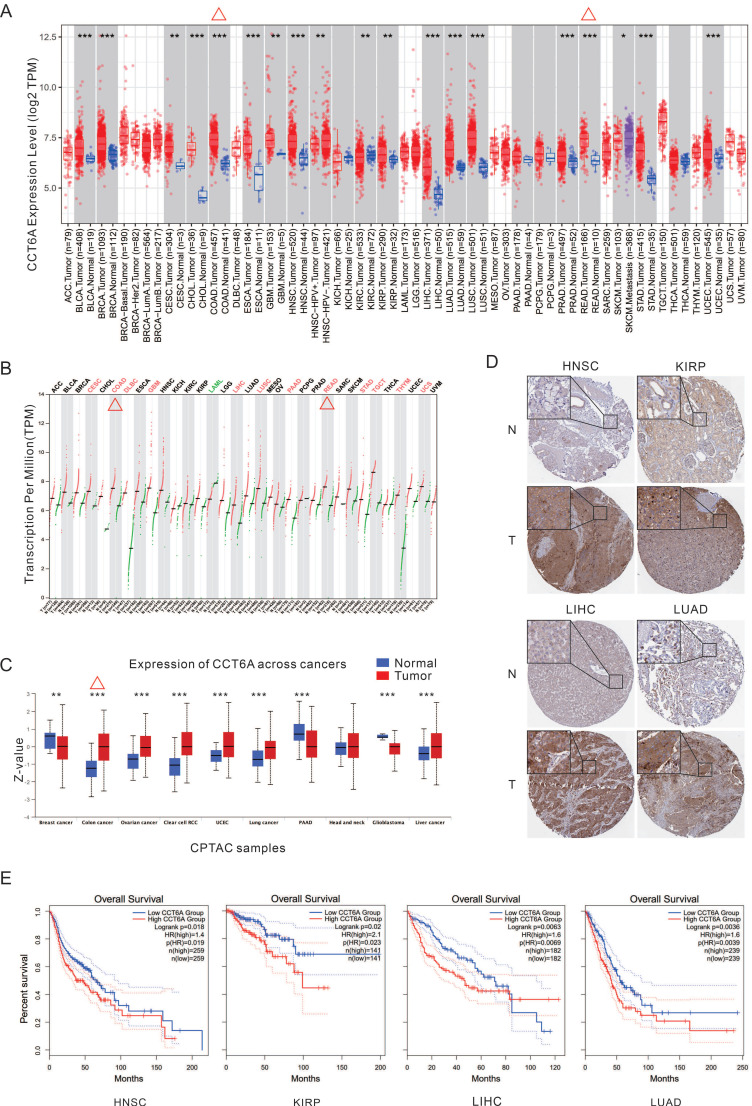
**Transcription and expression levels of CCT6A.** (**A**) Transcription levels of CCT6A in 33 tumor types in the TIMER2 database. (**B**) Transcription levels of CCT6A in 33 tumor types in the GEPIA database. (**C**) Expression levels of CCT6A in 10 tumor types in the CPTAC protein dataset of the UALCAN database. (**D**) IHC images of differential expressions of CCT6A protein in 4 tumors obtained from HPA database (https://www.proteinatlas.org/ENSG00000146731-CCT6A/tissue/salivary+gland#img; https://www.proteinatlas.org/ENSG00000146731-CCT6A/pathology/head+and+neck+cancer#img; https://www.proteinatlas.org/ENSG00000146731-CCT6A/tissue/kidney#img; https://www.proteinatlas.org/ENSG00000146731-CCT6A/pathology/renal+cancer#img; https://www.proteinatlas.org/ENSG00000146731-CCT6A/tissue/liver#img; https://www.proteinatlas.org/ENSG00000146731-CCT6A/pathology/liver+cancer#img; https://www.proteinatlas.org/ENSG00000146731-CCT6A/tissue/lung#img; https://www.proteinatlas.org/ENSG00000146731-CCT6A/pathology/lung+cancer#img). (**E**) Kaplan-Meier survival curves for patients with LIHC, LIHC, and THCA malignancies stratified by CCT6A expression. *P < 0.01, **P < 0.01 and ***P < 0.001.

By analyzing the TIMER database, CCT6A is significantly higher at the transcriptional levels in majority kind of cancers, such as CESC (cervical squamous cell carcinoma and endocervical adenocarcinoma), COAD (colon adenocarcinoma), GBM (glioblastoma multiforme), HNSC (head and neck squamous cell carcinoma), KIRP (kidney renal papillary cell carcinoma), LIHC (liver hepatocellular carcinoma), LUAD (lung adenocarcinoma), LUSC (lung squamous cell carcinoma), READ (rectum adenocarcinoma), STAD (stomach adenocarcinoma), and so on (**Figure 1A**). Meanwhile, the GEPIA database analysis also verified that CCT6A is highly expressed in multiple tumor tissues, in which COAD and READ included (**Figure 1B**). Furthermore, Analysis of CPTAC protein dataset in UALCAN database showed that CCT6A was significantly overexpressed in colon cancer, ovarian cancer, clear cell RCC (renal cell carcinoma), UCEC, lung cancer, and liver cancer (**Figure 1C**). In addition, the IHC images of four types of tumors (HNSC, KIRP, LIHC, LUAD) were downloaded from the HPA database, and these representative images showed that the staining intensity of CCT6A in tumor tissues was significantly higher than that in normal tissues (**Figure 1D**). Moreover, the Kaplan-Meier OS curves indicated that high CCT6A expression possess with a poor OS prognosis in HNSC (p = 0.019, HR = 1.4), KIRP (p = 0.023, HR = 2.1), LIHC (p = 0.0069, HR = 1.6), and LUAD (p = 0.0039, HR = 1.6) (**Figure 1E**). Generally, CCT6A is highly expressed at both transcription and expression levels in CRC (COAD combined READ).

### Expressional level and prognostic value of CCT6A in CRC

**Figure 2:**
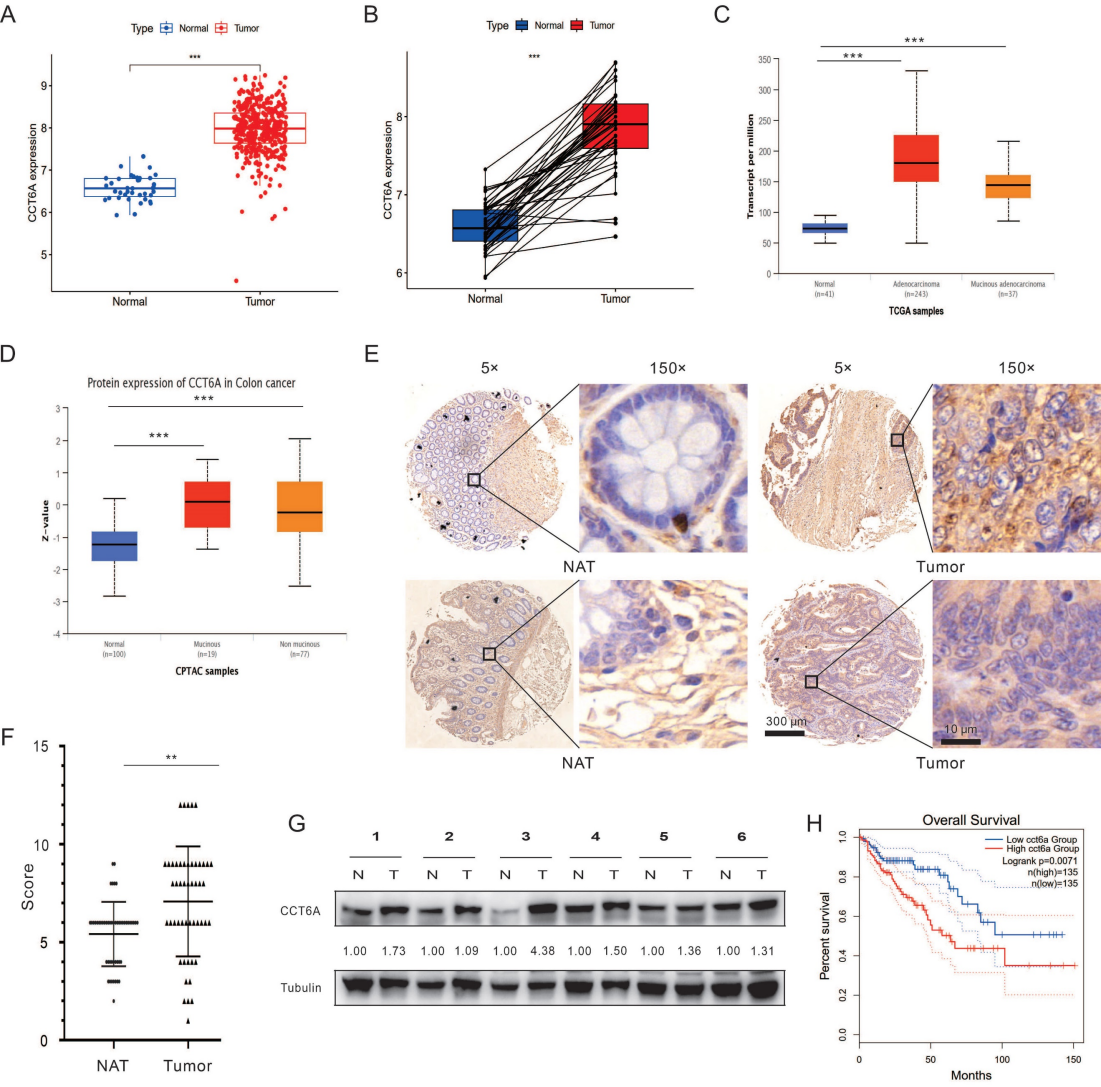
**Correlation between CCT6A expressions with CRC.** (**A**) Analysis of differences at transcriptional levels of CCT6A in normal and tumor tissues in the TCGA-COAD dataset. (**B**) Pairwise difference analysis of CCT6A at transcriptional levels in the TCGA-COAD dataset. (**C**) Analysis of differences in CCT6A transcriptional levels among normal tissue, colonic adenocarcinoma and non-mucinous adenocarcinoma in TCGA samples from UALCAN database. (**D**) Analysis of the difference of CCT6A protein levels among normal tissue, mucinous and non-mucinous cancer in CPTAC samples. (**E** and** F**) Representative images and quantification analysis of CCT6A level in a human CRC tissue microarray. (**G**) Total proteins from 6 pairs of fresh CRC tissue (T) and normal adjacent tissue (N) were extracted and performed immunoblotting assay with indicated antibodies. (**H**) Kaplan-Meier survival curves for patients with COAD stratified by CCT6A expression. **P < 0.01 and ***P < 0.001.

Then we paid our attention on the role of CCT6A in CRC. Primarily, the COAD dataset was downloaded from the TCGA database, and then subjected to R4.2.2 software to analyze the expression of CCT6A. As shown in **Figure 2A** and** B**, CCT6A was significantly higher expressed in tumor tissues compared with the paired normal tissues. Subsequently, we observed that expression of CCT6A was higher in both mucinous adenocarcinoma and adenocarcinoma than that in the normal tissues at both transcriptional level and protein level (**Figure 2C** and** D**). Meanwhile, similarly results were observed in READ (**Supplementary Figure 1A** and** B**). Then we conducted the IHC analyses with the CCT6A-specific antibody on a human CRC tissue microarray containing samples (50 CRC tissues and 50 matched intestine tissues). The results showed that CCT6A expression levels were higher in the cancer biopsies compared with the matched normal tissues (Mean of differences (T - N): 1.660, SD of differences: 3.121, 95% CI: 0.7731 to 2.547) (**Figure 2E** and **F**). Meanwhile, we collected 6 pairs of surgical specimens from patients with CRC, and the clinical information of the patients is shown in **Supplementary Table 1**. Compared with the adjacent normal tissues, the protein levels of CCT6A were higher in the tumor tissues of all tested cases (**Figure 2G**). Furthermore, we analyzed the correlation between CCT6A level and CRC overall survival, and high transcriptional level of CCT6A was associated with a worse prognosis for patients (**Figure 2H**).

### CCT6A positively modulates cell proliferation in CRC cells

**Figure 3:**
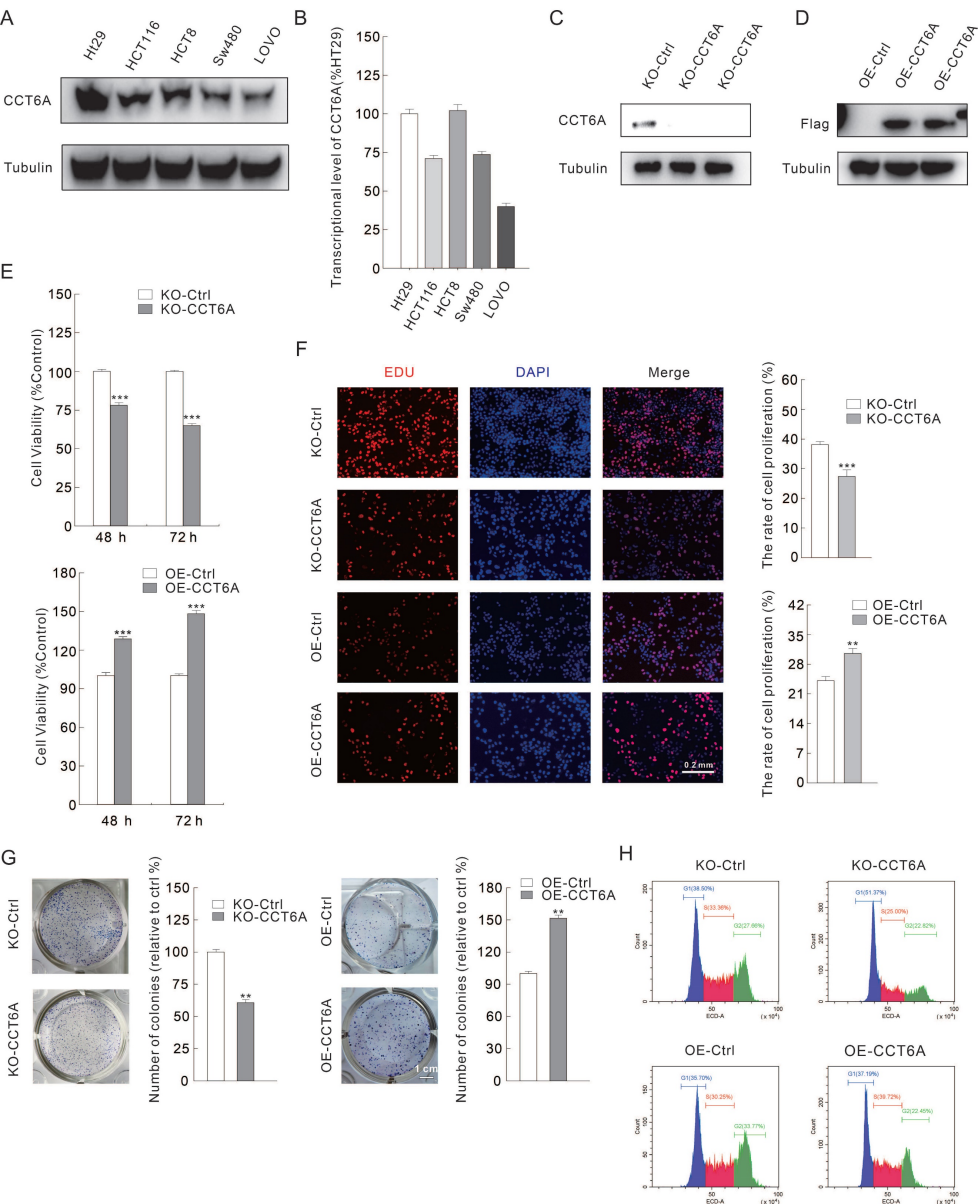
** CCT6A promotes cell proliferation in CRC cells.** (**A** and** B**) Immunoblotting and real-time PCR analysis of CCT6A expression in several ESCA cell lines. (**C** and **D**) KO-CCT6A HT29 cells and OE-CCT6A SW480 cells were constructed as described in Methods, and the knockout and overexpression efficacies were monitored by immunoblotting. (**E**) Cell viability was analyzed by MTS assay. (**F** and **G**) colony growth assay (scale bars = 1 cm) and EDU staining assay (scale bars = 0.2 mm) were performed to detect the cell proliferation activity. (**H**) Indicated cells were collected, stained and performed flow cytometry to detect cell cycle. **P < 0.01, and ***P < 0.001.

As CCT6A is highly expressed and high CCT6A expression possesses a poor OS prognosis in CRC, we then verified whether CCT6A plays a role in regulating cell proliferation in CRC cells. Several CRC cell lines were cultured, and then the total RNAs and proteins were extracted. Utilizing immunoblotting and RT-PCR, we found that CCT6A was highly expressed in HT29 and HCT8 cells, and lowly expressed in SW480 and LOVO cells at both protein level and mRNA level (**Figure 3A** and** B**). Then, HT29 and SW480 cells were selected for the subsequently study. CCT6A knockout HT29 cells were constructed as described in the methods, and the efficacy was confirmed by immunoblotting assay (**Figure 3C**). On the contrary, Flag-CCT6A plasmids were transfected into SW480 cells, and the transfection effect was verified by monitoring the Flag expression (**Figure 3D**). Compared to their corresponding control cells, knockout of CCT6A (KO-CCT6A) reduced the cell viability at both 24 h and 48 h time points, while its overexpression (OE-CCT6A) led to completely opposite results (**Figure 3E**). In colony growth assay, the colony formation in CCT6A-KO was significantly reduced to about 61% compared to the control group, while OE-CCT6A increased the colony formation to approximately 152 percent (**Figure 3F**. The positive correlation between CCT6A and cell proliferation was confirmed by EDU staining assay, as the EDU positive cell percentage in KO-CCT6A group was lower, and OE-CCT6A group was higher when compared to their corresponding control group, respectively (**Figure 3G**). Moreover, flow cytometry was used to detect the cell cycle changes in the CCT6A trans-genetic cells. The results showed that the proportion of S phase cells in the KO-CCT6A group was significantly lower than that in the control group (33% VS 25%), while the overexpression group showed the opposite trend (30% VS 40%) (**Figure 3H**). Therefore, CCT6A plays a positive role in regulating cell proliferation in CRC cells.

### CCT6A involves in regulating cell migration in CRC cells

**Figure 4:**
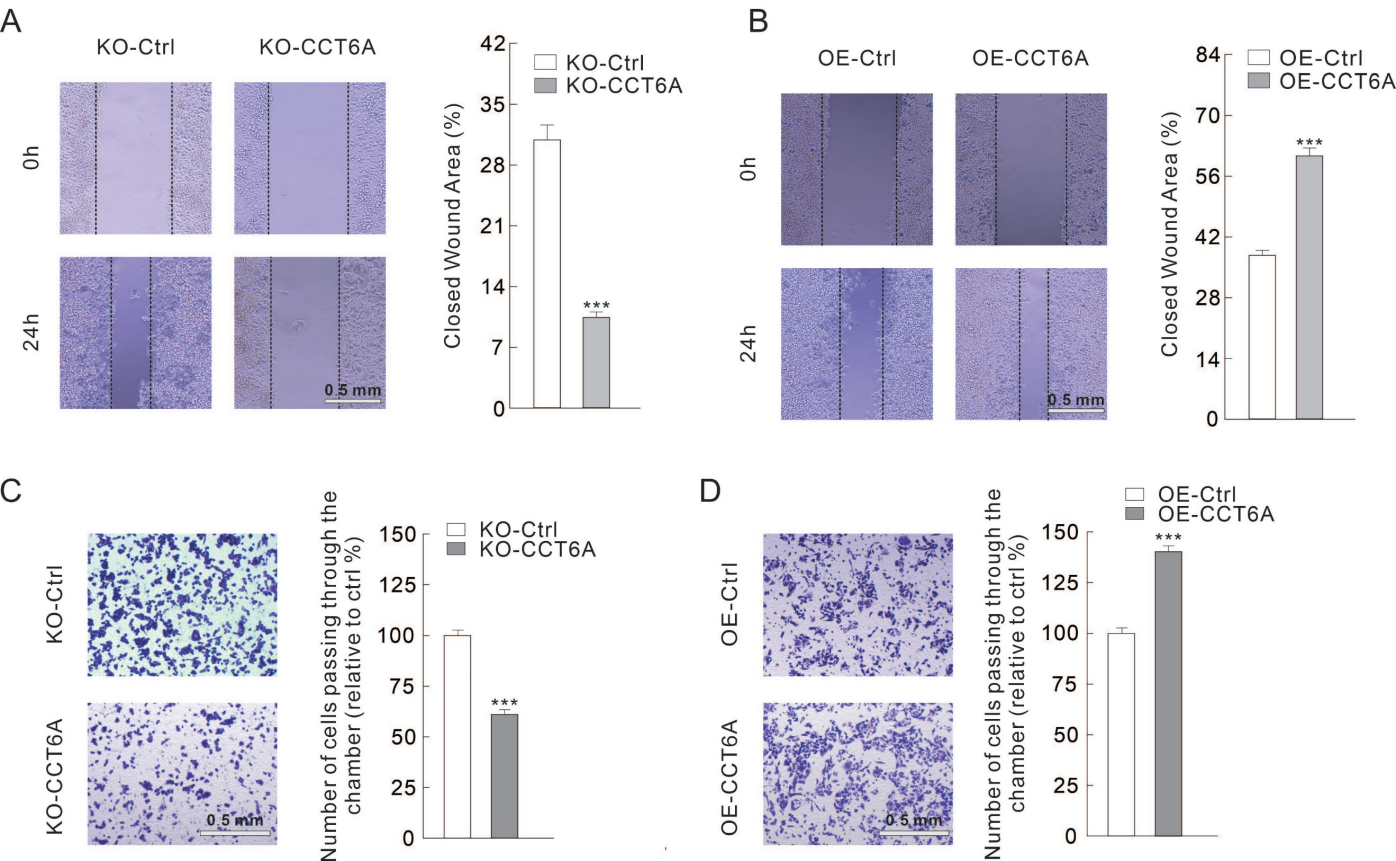
**CCT6A promotes cell migration in CRC cells.** (**A** and** B**) Wound healing assay was performed in indicated cells, and pictures were taken at 0 and 24 h time points (scale bars = 0.5 mm). (**C** and** D**) Cell invasion was monitored by transwell assay with indicated cells (scale bars = 0.5 mm). ***P < 0.001.

Then we evaluated whether CCT6A could participate in regulating cell migration in CRC cells. Utilizing wound healing assay, we observed that the closed wound area in KO-CCT6A cells was significantly smaller than that in the control cells after 24 h culture (31% VS 10%) (**Figure 4A**). On the contrary, OE-CCT6A enhanced the cell migration as the unhealed wound area was less in the OE-CCT6A cells (38% VS 61%) (**Figure 4B**). Moreover, the cell invasion activities were evaluated by Transwell assay under Matrigel pre-incubation condition. The results showed that the cells passing through the chamber were less in the KO-CCT6A group (about 61% to control), while OE-CCT6A increased the cell number crossed the chamber (about 140% to control) (**Figure 4C** and** D**). In conclusion, CCT6A positively regulates cell migration/invasion in CRC cells.

### Correlation between CCT6A and TIME in CRC

Recent years, as TIME plays an increasingly important role in cancer research and therapy, it has attracted the attention of many researchers 19. The COAD and READ datasets (combined as CRC hereafter) from TCGA database were downloaded, and the correlation between CCT6A expressions (cut-off: 50%) with TIME was then analyzed by R4.2.2 software. By scoring the content of stromal cells and immune cells in the TIME, it was found that the scores of stromal cells, immune cells and the ESTIMATE were lower in the CCT6A high expression group (**Supplementary Figure 2A**). Utilizing analysis with CIBERSORT algorithm, several immune cells amount were different in CCT6A high and low expression groups, such as T cells regulatory (Tregs) were more in CCT6A low expression group, while Macrophages (especially M1) were increased in the high expression group (**Supplementary Figure 2B**). Moreover, with the help of immune cell correlation analysis by R4.2.2 software, M0 (p =0.011, R =0.16) were significantly positive correlated with the expression of CCT6A (**Supplementary Figure 2C** and **D**). On the contrary, Tregs (p = 1.4e-08, R = -0.35), B cells memory (p = 0.028, R = -0.14), plasma cells (p = 0.039, R = -0.13) and mast cells activated (p = 0.041, R = -0.13) showed a negative correlation with CCT6A (**Supplementary Figure 2C** and **D**). These cells are closely related to the occurrence, development and prognosis of tumors 20, 21. For example, the decrease or loss of B memory and plasma cells will reduce the anti-cancer immune response, which is correlated with cell carcinogenesis and poor prognosis 22.

Then we analyzed the correlation between immune checkpoint and CCT6A in CRC datasets, and the results showed that CCT6A was positively correlated with immune checkpoints TNFSF18, CD44, TNFSF4, while negatively correlated with TNFSF14, CD27, LGALS9 (**Supplementary Figure 2E** and** F**). Furthermore, the clinical data related to immunotherapy from TCIA was analyzed, and the low score indicated poor immunotherapy efficacy. In CTLA4 and PD1 negative, single positive and all positive conditions, patients in the CCT6A high expression group had a worse therapeutic effect than those in the low expression group, indicating that CCT6A was significantly correlated with immunotherapy efficacy (**Supplementary Figure 2G**).

### CCT6A-associated biological processes analysis

To investigate the molecular mechanism and signaling pathways of CCT6A in carcinogenesis, we performed GO enrichment analysis using R4.2.2 software with CRC datasets from TCGA database. It was found that CCT6A is related with a variety of immune responses, such as immunoglobulin production, mediator of immune responses, and humoral immune responses (**Supplementary Figure 3A** and** B**). KEGG enrichment analysis showed that CCT6A was related to serotonergic synapse, taste transduction and MAPK signaling pathway (**Supplementary Figure 3C** and** D**). Noteworthy, CCT6A was found to associate with MAPK signaling, estrogen signaling, cell adhesion molecules, all of which played essential roles in carcinogenesis (**Supplementary Figure 3C** and** D**). Additionally, GSEA functional analysis showed that CCT6A was related to DNA replication, non-coding RNA processing, as well as ribosome biogenesis, suggesting that CCT6A was related to cell cycle, nucleotide excision repair and RNA degradation (**Supplementary Figure 3E**).

**Figure 5:**
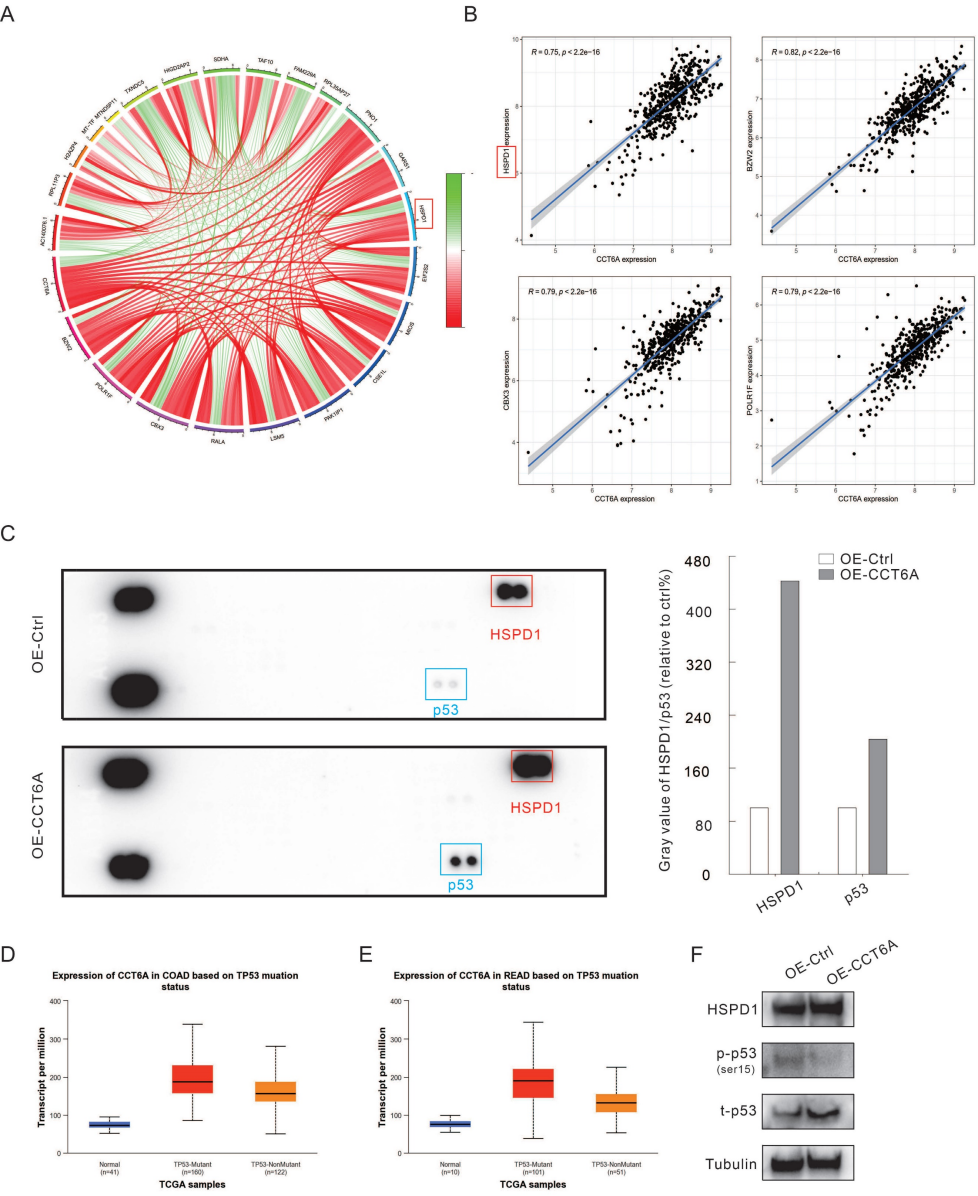
**CCT6A promotes HSPD1 and P53 expression.** (**A**) Circle diagram of CCT6A co-expression analysis (TCGA-COAD and READ datasets). (**B**) Co-expression analysis of CCT6A with HSPD1, BZW2, CBX3, and POLR1F (TCGA: COAD and READ datasets). (**C**) Cell lysates were extracted from OE-Ctrl and OE-CCT6A SW480 cells, and then performed phosphor-kinase array assay according to the instructions. The blots were analyzed by Image J software. (**D** and** E**) The relationship between transcriptional level of CCT6A and P53 mutant status in COAD and READ datasets from UALCAN database were analyzed. (**F**) Cell lysates were subjected to immunoblotting with indicated antibodies.

Co-expression analysis using R4.2.2 in CRC datasets showed that the expression of CCT6A was positively correlated with HSPD1 (HSP60), BZW2, CBX3, and POLR1F (**Figure 5A** and** B**). Furthermore, we performed the human phosphor-kinase array kit to identify the molecules affected by CCT6A overexpression. The results showed that the expression of HSPD1 was significantly increased (about 4 times to Ctrl), which further confirmed that CCT6A was positively correlated with HSPD1 (**Figure 5C**). And former studies indicated that abnormal expression of HSPD1 is associated with tumor cell metastasis and drug resistance 23-25. At the same time, P53 was also found to obviously increase in the OE-CCT6A group (**Figure 5C**). Analysis of TCGA samples in the UALCAN database showed that in normal tissues, P53-mutant and P53-nonmutant tumor tissues, CCT6A transcript levels were the highest in the P53 mutant group, and both were higher than normal tissues, suggesting that CCT6A levels were positively correlated with P53 mutation status (**Figure 5D** and** E**). Although it was originally believed to negatively regulate tumorigenesis, mutation of p53 was generally observed in cancer cells, which play a role in promoting cell proliferation and resisting therapy 26, 27. We then carried out immunoblotting assay, and overexpression of CCT6A increased the protein levels of HSPD1 and p53 (**Figure 5F**). However, OE-CCT6A failed to increase the phosphorylated-P53 (ser15) level, indicating that the activity of P53 was lower in these cells (**Figure 5F**). These results indicated that HSPD1 and p53 may involve in the CRC tumorigenesis regulated by CCT6A.

## Discussion

Previous studies have suggested that CCT6A participates in several biological processes in a variety of tumors, and it has been mentioned to have certain prognostic value and clinical significance. As for the CRC, CCT6A may play a role in affecting liver metastasis of CRC 11, and some reports have suggested that CCT6A is related to the occurrence of immune infiltration 28. Here, we explored the expression level of CCT6A from the perspective of pan-cancer and multi-omics, analyzed the immune value of CCT6A, and preliminary verified its role in regulating cell proliferation/migration in CRC cells.

Due to the different sources of data in public databases, there are differences in the quality of the collected data and the subtle conditions of screening, which may affect the results of the analysis. For instance, the expression patterns of CCT6A in cancers were inconsistent when comparing the results from different databases. Even so, CCT6A was found to highly expressed in CRC at both transcriptional and protein levels. Meanwhile, the CRC tissue microarray and CRC patients' tissues verified that CCT6A levels were higher in the tumor tissues than in the adjacent normal tissues. Subsequently, the prognostic value of CCT6A was analyzed by GEPIA, and it was found that CCT6A is a high risk factor in a variety of tumors. The overall survival of patients with CCT6A high expression is poor, suggesting that CCT6A has a certain clinical prognostic value, especially in CRC.

Solid tumors are in a complex microenvironment, including stromal cells and immune cells, and the tumorigenesis is closely related to these factors. The expression of CCT6A was found to negatively correlation with the content of stromal cells and immune cells. Tregs are a small group of immune cells that inhibit excessive immune responses and maintain immune homeostasis 29. Although various studies have maintained that high Tregs infiltration in TME inhibits effective anti-tumor immune response, leading to tumor initiation and progression 30, some studies have suggested that Tregs infiltration in some part of tumors such as CRC can inhibit excessive inflammation, one of the factors leading to tumorigenesis, therefore playing a protective role 31-33. Besides Tregs, activated mast cells, plasma cells and memory B cells were also negatively correlated with CCT6A expression, all of which are involved in regulating tumorigenesis 34-36. On the contrary, both M1 and M2 (although p > 0.05) macrophages amounts were found to positively associate with CCT6A expression, while the former usually play an anti-tumor role, and the later promote the occurrence and metastasis of tumors 37. However, some studies have suggested that M1, while playing a pro-inflammatory role, also triggers the expansion and self-renewal of cancer stem cells, thereby promoting tumorigenesis 38, 39. Therefore, more exploration is needed to understand the role of macrophages in CRC tumorigenesis. Immune checkpoint blockade is one of the important forms of immunotherapy, and the representative drugs including anti-PD1 and anti-CTLA4 40. Analysis of the clinical data of anti-PD1 and anti-CTLA4 application showed that the therapeutic effect in the CCT6A-high expression group was worse than that in the low expression group. Immune checkpoint correlation analysis also showed that TNFSF18, CD44 and TNFSF4 were positively correlated with the expression of CCT6A. These observations indicated that CCT6A possessed immune value, and potentiality as a target for immunotherapy in CRC.

Although the multi-omics bioinformatics analysis indicated that CCT6A could function as a biomarker for CRC and had prognostic value, some deficiencies still need to be covered by experimental investigations, such as the modulation of CCT6A on cell proliferation/migration and the intrinsic mechanism in CRC. Here, we generated CCT6A knockout cells in HT29 by CRISPR-Cas9, and overexpressed by transfecting plasmids in SW480 cells. Utilizing these cells, we demonstrated that CCT6A positively regulate cell proliferation and cell migration. We also preliminary explored the downstream molecules affected by CCT6A overexpression by human phosphor-kinase array assay in SW480 cells, and HSPD1 and p53 were found to robustly increased in OE-CCT6A group. HSPD1 has been reported to involved in many processes of cell activates, such as protein folding, apoptotic and necrotic cell death 41, 42. Ghosh *et al.* demonstrated that deprivation of HSPD1 activated p53-dependent and mitochondrial apoptosis 43. P53 functions as a tumor suppressor by maintaining genomic stability and controlling apoptosis as well as cell cycle 27, 44, 45. Its deletion and mutation were generally emerged in tumor cells, which led to cell out of control 46. Here, we found OE-CCT6A increased the level of total P53, but not the phosphorylated-P53 (ser15). However, validation on whether and how HSPD1/p53 involves in the CRC tumorigenesis regulated by CCT6A need to be investigated in the future study.

## Conclusion

In summary, the presented data showed that CCT6A was highly expressed in numerous tumor tissues and was closely related to the prognosis of various tumors. As for the CRC, bioinformatics analysis and experimental validation indicated that CCT6A levels were higher in the tumor group than that in the normal group. Furthermore, overexpression of CCT6A promoted cell proliferation and migration, while its knockout presented the opposite phenotypes. These results revealed the importance of CCT6A expression in the discovery and prognosis, and provided a theoretical basis for CCT6A as potential therapeutic target for CRC.

## Supplementary Material

Supplementary figures and table.

## Figures and Tables

**Table 1:** Primer sequences for amplification.

Gene	Primer	Nocleotides
CCT6A	Forward (5′ → 3′) Reverse (5′ → 3′)	CTGAAACAGGCGGATCTCTACA CCCTGTCCATCTCTCTGCTTAC
β-Actin	Forward (5′ → 3′) Reverse (5′ → 3′)	GCCTGACGGCCAGGTCATCAC CGGATGTCCACGTCACACTTC
