# Supplementary material for: CCT6A functions as promising diagnostic biomarker and promotes cell proliferation in colorectal cancer
Source: J Cancer. 2024 Sep 16;15(18):5897–909. doi: 10.7150/jca.98901 (PMC11493007; doi:10.7150/jca.98901)
Supplement: Supplementary file 1 — Supplementary figures and table. [file jcav15p5897s1.pdf]

Supplementary Figure Legend:

Supplementary Figure 1. Transcription levels of CCT6A in READ. (**A**) Analysis of differences at transcriptional levels of CCT6A in normal and tumor tissues in TCGA-READ from UALCAN database. (**B**) Analysis of differences in CCT6A transcriptional levels among normal tissue, rectal adenocarcinoma and non-mucinous adenocarcinoma in TCGA samples from UALCAN database. ***P < 0.001.

Supplementary Figure 2. Correlation analysis of CCT6A and immune value. (**A**) Analysis of correlation in TIME between CCT6A high- and low-expression groups. (**B**) R4.2.2 was utilized to analyze the association between the immune cell amount and CCT6A expression. (**C** and **D**) Immune cell correlation with CCT6A expression was analyzed by TIME and R4.2.2, respectively. (**E** and **F**) Correlation analysis between CCT6A and immune checkpoints by TCGA-COAD and READ datasets. (**G**) Public data from TCIA was utilized to analyze the association between immunotherapy effect and CCT6A expression in CRC. *P < 0.01, **P < 0.01 and ***P < 0.001.

Supplementary Figure 3. GO, KEGG, and GSEA enrichment analysis of CCT6A. (**A** and **B**) Bar plot and bubble plot of CCT6A by GO enrichment analysis (TCGA-COAD and READ datasets). (**C** and **D**) Bar graph, bubble plot of CCT6A by KEGG enrichment analysis (TCGA-COAD and READ datasets). (**E**) Functional and pathway analysis of CCT6A by GESA (TCGA-COAD and READ datasets).

Supplementary Figure 1

A

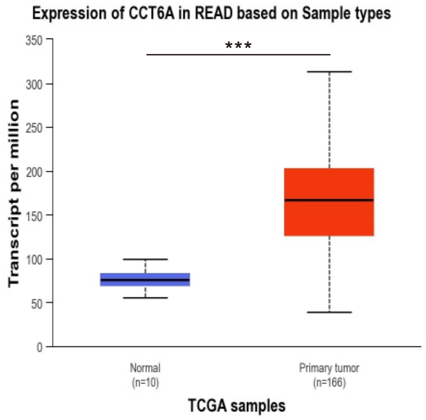

B

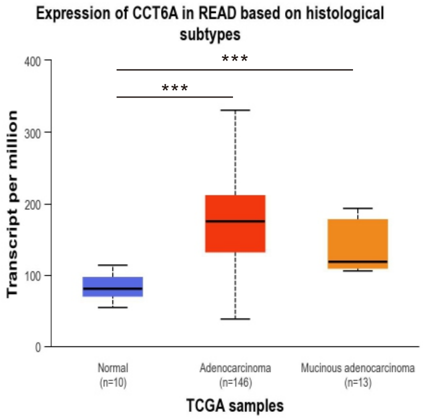

Supplementary Figure 2

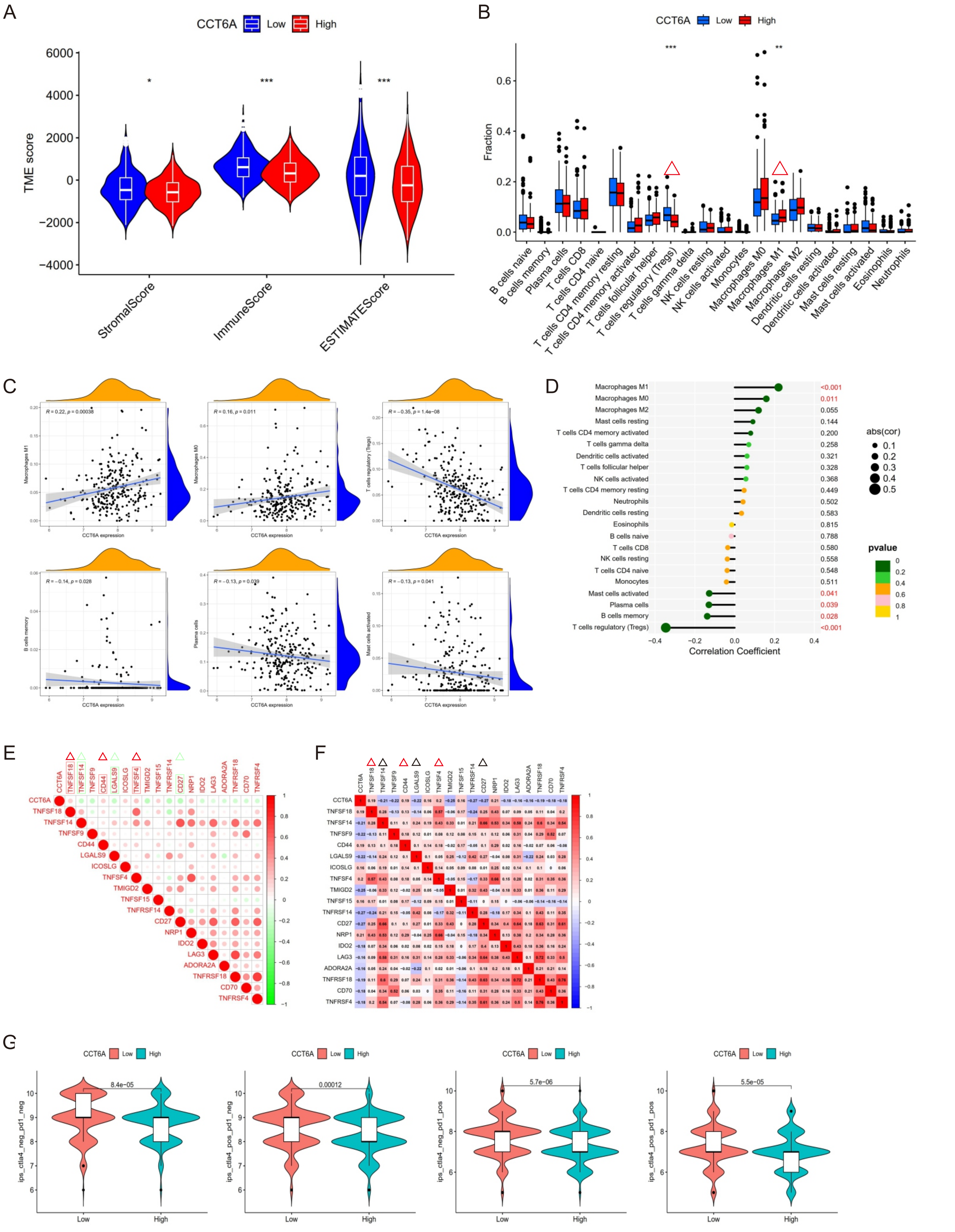

Supplementary Figure 3

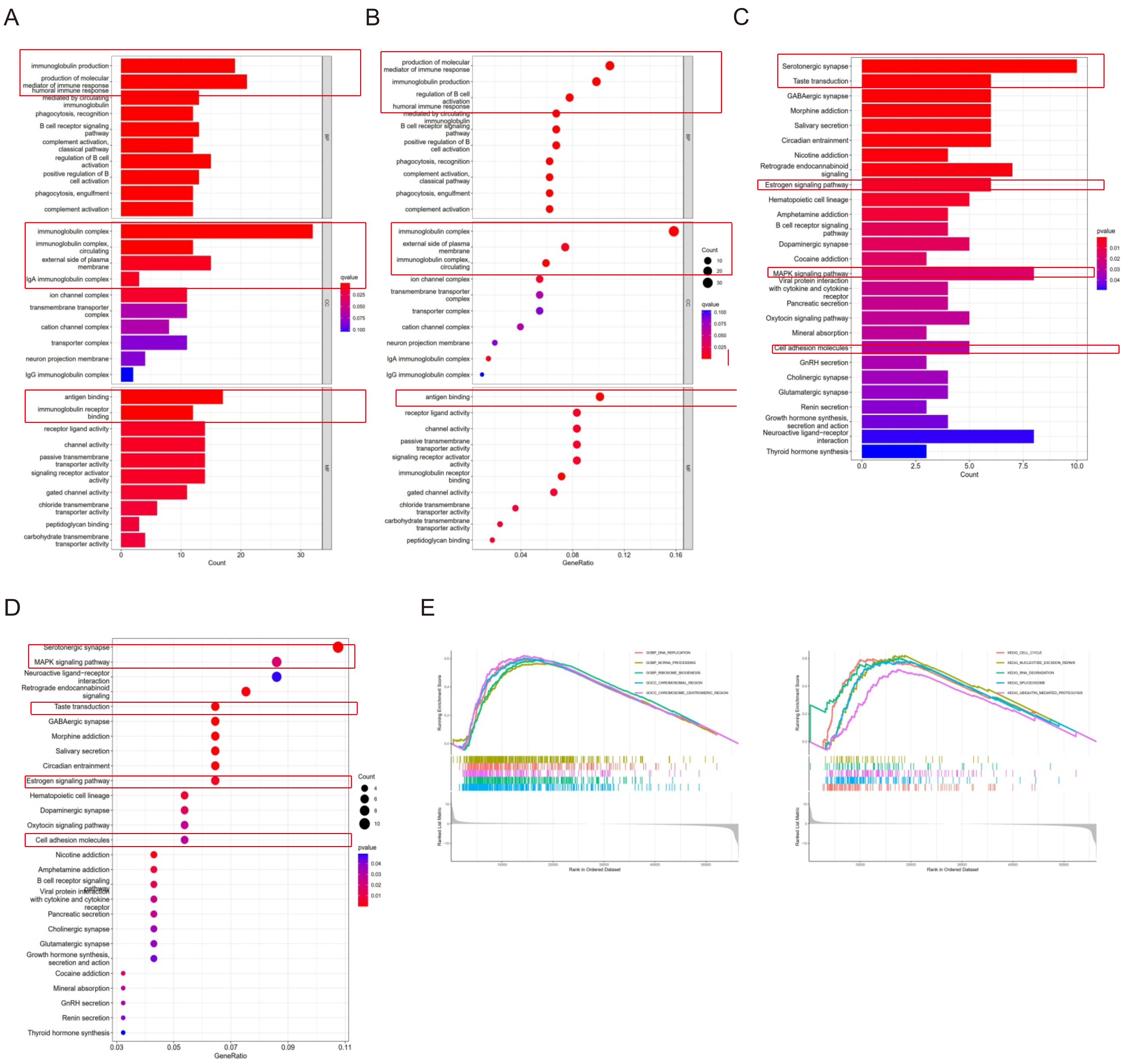

Supplemental Table 1. Patients information.

The table listed the patients' information of the tumor samples used in the current study, whom were underwent surgical treatment at the Second Hospital of Lanzhou University from 2021 to 2022.

Sample Number	ID Number	Sex	Age	Preoperative diagnosis	Pathology Diagnosis (postoperative pathology)	Complications	pTNM	Stage	Nerve invasion or not	Vascular invasion or not
#1	1191596	F	88	Colon neoplasm	Right-side colon infiltrative ulcer type - in poorly differentiated adenocarcinoma	None	T4bN2bM0	IIIC	Yes	Yes
#2	1195476	M	66	Sigmoid colon malignant tumor	Sigmoid colon ulcerative moderately differentiated adenocarcinoma	None	T3N0M0	IIA	Yes	Yes
#3	1182659	F	76	Malignant tumor of the colon	Invasive ulcer type - in poorly differentiated adenocarcinoma of the colon	None	T3N2aM0	IIIB	Yes	Yes
#4	1200432	M	52	Malignant tumors of the colon	Infiltrating ulcerative moderately differentiated adenocarcinoma of the colon	None	T3N0M0	IIA	Yes	Yes
#5	1205577	M	54	Sigmoid colon malignant tumor; Metastasis of colon cancer to the liver	Ulcerated moderately to poorly differentiated adenocarcinoma of the sigmoid colon	None	T3N1cM1a	VIA	Yes	Yes

#6	1201511	M	56	Malignant tumor of the colon	Moderately to poorly differentiated mucinous adenocarcinoma of the colon	None	T4bN2aM0	IIIC	Yes	Yes
